# Ultrasound enhances the recycling process and mechanism of lithium from spent LiFePO_4_ batteries by *Acidithiobacillus ferrooxidans*

**DOI:** 10.1038/s41598-025-08952-w

**Published:** 2025-07-08

**Authors:** Shaoliang Zhang, Qin Chen, Weihua Gu, Jianfeng Bai

**Affiliations:** School of Resources and Environmental Engineering, Shanghai Polytechnic University, No. 2360, Jinhai Road, Pudong New District, Shanghai, 201209 China

**Keywords:** Bioleaching, Decommissioned lithium-ion power batteries, Leaching mechanism, Environmental impact, Environmental microbiology

## Abstract

In this study, the ability of *Acidithiobacillus ferrooxidans* to oxidize Fe^2+^ to Fe^3+^ and recover battery black powder was investigated, establishing a system for leaching decommissioned lithium iron phosphate battery black powder from *A. ferrooxidans*. Black powder reduced the consumption of reagents and subsequent pressure for treating iron-bearing minerals using the iron source in waste LiFePO_4_ batteries. This study used ultrasonic waves to remove impurities on the surface and cracks in battery black powder, hindering the dissolution layer and enhancing the leaching effect through a cavitation reaction and microbial activation to promote the leaching process. A filter bag experiment was designed using the selective permeability of filter bags to investigate whether the leaching mechanism of *A. ferrooxidans* lithium iron phosphate is contact or non-contact. Under optimal leaching conditions, the lithium leaching rate reached 99.7%, and the leaching time was reduced from 7 to 5 days, achieving efficient leaching of lithium. The filter bag experiment concluded that *A. ferrooxidans* leaching of lithium iron phosphate was mainly a contact leaching mechanism.

## Introduction

Recently, there has been a significant surge in the widespread use of portable electronic devices, electric vehicles (EVs), and energy storage systems, which has led to an increased demand for lithium-ion batteries^1–7^. This surge in demand inevitably results in a corresponding increase in battery retirements. According to Abdalla et al.^5^, dynamic-model Monte Carlo simulations predict that by 2030, China’s retired power batteries from new energy vehicles will reach 7.16 million sets, weighing approximately 6.55 million tons. Consequently, the efficient and eco-friendly disposal and recycling of retired power batteries necessitate urgent measures.

Recycling retired lithium-ion batteries primarily involves pre-treatment, extraction, and recycling of valuable metals^8^. Zhang et al.^9^ used a shear crusher to fragment waste lithium-ion batteries into pieces, which were subsequently crushed into a black powder via an impact crusher. Similarly, Xu et al.^10^ employed shredders, shakers, and vibrating screens to recycle waste lithium-ion batteries, yielding a light product, a synthetic resin separator, and a mixture of heavy products comprising aluminum foil, copper foil, LiCoO_2_, and graphite.

The extraction and recovery of valuable metals include pyrometallurgy, hydrometallurgy, and microbial metallurgy^11,12^. Tang et al.^13^ converted LiCoO_2_ into CoO and Li_2_CO_3_ by mixing crushed LiCoO_2_ powder with graphite under specific conditions, enabling Li and Co separation. Sun et al.^14^ subjected LiCoO_2_ batteries to pyrolysis under a vacuum at 600 °C for 30 min, leading to the detachment of almost all LiCoO_2_ powder from the aluminum foil and facilitating subsequent Li and Co recovery. Lombardo et al.^15^ employed calcination to recover ternary lithium-ion batteries, Li(Ni_x_Mn_y_Co_z_)O_z_, yielding the final calcination products CoO, Co_3_O_4_, NiO, Mn_3_O_4_, MnO_2_, Li_2_O, and Li_2_CO_3_.

Hydrometallurgy is currently the most widely used method for recycling waste lithium ions^16,17^. Gu et al.^18^ recovered lithium, iron, and aluminum from lithium iron phosphate using alkali leaching followed by acid leaching. Zhuang et al.^19^ used a mixture of phosphoric and citric acids to recover valuable metals from ternary lithium-ion batteries (LiNi_0.5_Co_0.2_Mn_0.3_O_2_) and achieved high leaching rates of Li, Ni, Co, and Mn under optimal conditions. Li et al.^20^ employed a sulfuric acid–hydrogen peroxide leaching system to recover lithium from retired lithium iron phosphate power batteries, using sulfuric acid as the leaching agent and hydrogen peroxide as the oxidant. Gong et al.^21^ selectively leached lithium from lithium iron phosphate using H_2_O_2_ and sodium bisulfate as the oxidant and leaching agent, respectively. Additionally, Li et al.^22^ compared the use of H_2_SO_4_, HCl, and citric acid to recover LiCoO_2_ and demonstrated that citric acid exhibited higher leaching rates for Co and Li than H_2_SO_4_ and HCl. Esmaeili et al.^23^ investigated ultrasound-assisted organic acid leaching for ternary lithium-ion batteries using organic acids from lemon juice and H_2_O_2_ as leaching agents.

Microbial metallurgy employs microorganisms (bacteria and fungi) or metabolites to recover valuable metals from metal-containing minerals, electronic waste, and sewage sludge^2,4,24,25^. Naseri et al.^26^ used *Acidithiobacillus ferrooxidans* to recover Li, Co, and Mn from lithium-ion batteries using one- and two-step leaching methods and achieved high leaching rates under optimal conditions. Roy et al.^27^ reduced leaching time by replacing the bacterial solution during the leaching process using *A. ferrooxidans* bioleaching in recovering valuable metals from retired ternary lithium-ion batteries. Liao et al.^28^ studied the role of reducing iron (Fe^2+^ and Fe^0^) in the recovery of a LiCoO_2_ system using a mixed strain of *Thermophilic Acidophilic Thiobacillus* and *Thermophilic Thiobacillus*. They suggested avoiding reducing agents that hinder bacterial growth when assisting microbial leaching processes. Do et al.^29^ combined microbial metallurgy with LIB regeneration and demonstrated its industrial development prospects. Wu et al.^30^ investigated the effects of different energy substances and bacterial oxidation products on microbial metallurgy by enhancing the leaching rates of Li^+^ and Co^2+^ using mixed cultures of A. t and A. f bacteria.

Despite the environmental benefits of microbial metallurgy compared to pyrometallurgy and hydrometallurgy, microbial metallurgy often encounters challenges, such as lengthy processing cycles and slow reaction efficiency^31–34^. To address these issues, this study proposed ultrasonic-enhanced microbial metallurgy, which uses a mixture of positive and negative electrodes (black battery powder) from retired lithium iron phosphate power batteries. Using *A. ferrooxidans*, we aimed to oxidize Fe^2+^ to Fe^3+^ for energy generation, produce H^+^, oxidize Fe^2+^ in lithium iron phosphate (LiFePO_4_), dissolve lithium in acidic environments, and achieve efficient lithium recovery. Moreover, a detailed analysis of the adsorption kinetics of the reaction was conducted to explore the mechanism by *A. ferrooxidans* in leaching lithium iron phosphate, thus providing a theoretical foundation for further studies.

## Materials and methods

### Black powder of spent LiFePO_4_ power batteries

The black powder sample was obtained from spent LiFePO_4_ power batteries provided by a recycling enterprise in Suzhou, China. The primary constituents were lithium, iron, and graphite (Table 1).

**Table 1:** The primary constituents of the black powder sample.

Constituent	Li	Fe	P	Cu	Al	C
Mass fraction (%)	3.56	36.79	14.38	1.72	0.16	43.39

### Microorganisms and growth

The *A. ferrooxidans* were isolated, domesticated, and cultivated in our laboratory. The culture medium used in the bioleaching process of *A. ferrooxidans* termed the improved 9K culture medium, comprised (NH_4_)_2_SO_4_ (3 g/L), KCl (0.1 g/L), K_2_HPO_4_ (0.5 g/L), MgSO_4_·7H_2_O (0.5 g/L), and Ca(NO_3_)_2_ (0.01 g/L), except for FeSO_4_ found in the traditional 9K medium. The preparation involved sequentially adding the above ingredients to 1000 mL of deionized water, followed by high-temperature sterilization and stirring until complete dissolution. Subsequently, the pH of the culture medium was adjusted to 2.0 using H_2_SO_4_ (5 mol/L), resulting in an improved 9K culture medium^35,36^. The Fe^2+^ necessary for *A. ferrooxidans* growth was provided by LiFePO_4_.

### Bioleaching experiments

#### Leaching influencing factors experiment

The blank control experiment involved adding battery black powder to deionized water with the pH adjusted to 2.0, using H_2_SO_4_. Modified 9K medium (pH 2.0) was used in conical flasks inoculated with *A. ferrooxidans* solution. The leaching rate of the LiFePO_4_ black powder was monitored every 24 h to differentiate between *A. ferrooxidans* bioleaching and acid leaching and to determine the leaching cycle length. Exploratory factors included FeSO_4_ addition, solid–liquid ratio, temperature, initial pH, oscillation rate, and inoculation amount^36^.

#### Ultrasonic-enhanced leaching experiment

**Fig. 1:**
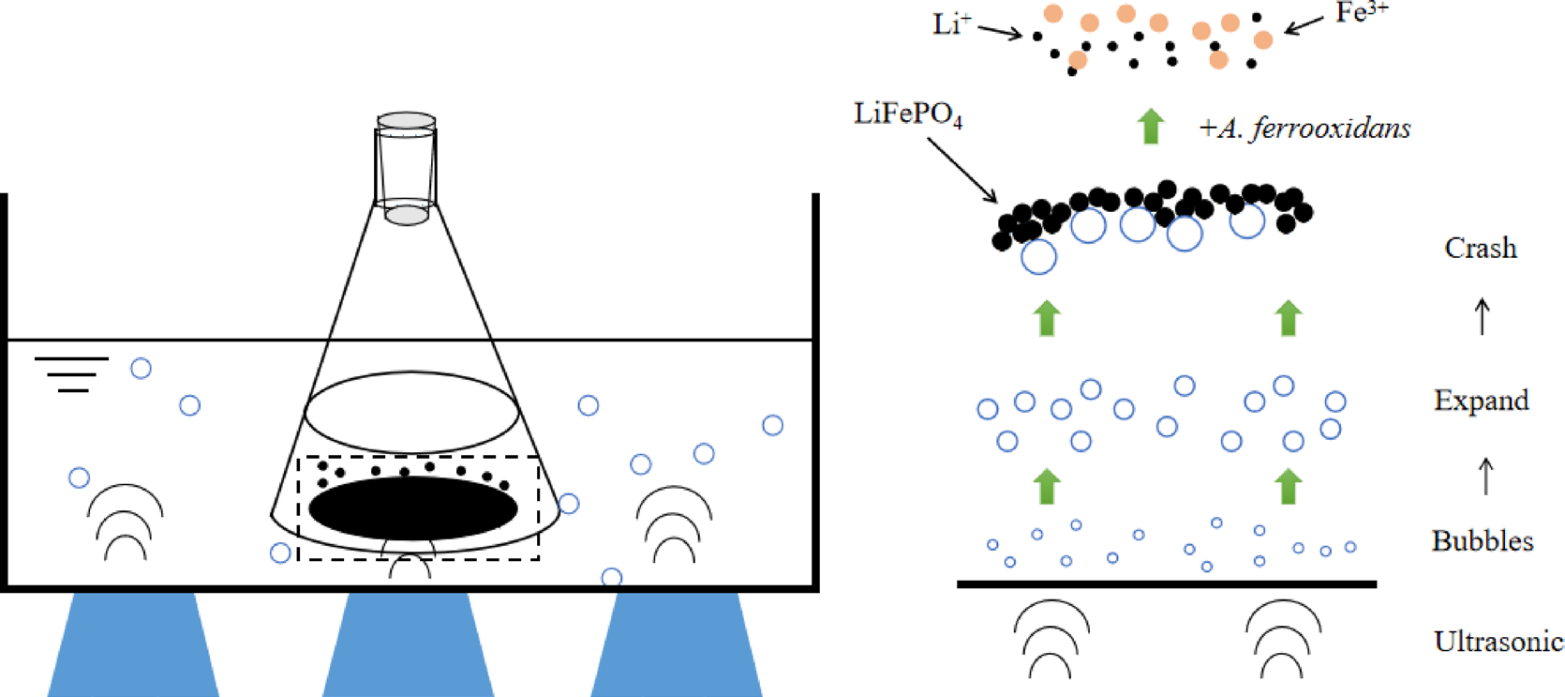
Mechanism of ultrasound enhanced leaching of black powder of LiFePO_4_ battery using *Acidithiobacillus ferrooxidans.*

The use of ultrasonic waves to reinforce *A. ferrooxidans* bioleaching of LiFePO_4_ battery black powder aimed to improve the leaching rate. The process utilized cavitation effects, as shown in Fig. 1, in which ultrasonic strengthening generates and expands small bubbles in the leaching solution, ultimately rupturing and creating energy differentials that promote biological leaching^37^.

##### Ultrasound frequency

In the ultrasonic intensification leaching process, an ultrasonic frequency that is too low leads to insufficient ultrasonic intensification. In contrast, an ultrasonic frequency that is too high directly affects the activity of *A. ferrooxidans* or even leads to the death of *A. ferrooxidans*. Therefore, leaching experiments were designed using different ultrasonic frequencies. The *A. ferrooxidans* solution, cultured to the log growth stage after domestication, was inoculated into the modified 9K medium to bioleach LiFePO_4_ black powder at different inoculums. The leaching conditions for *A. ferrooxidans* were selected as the optimal conditions derived from experiments in “Materials and methods” to “Results and discussion” sections. On the first day of the experiment, the free *A. ferrooxidans* in the system adsorbed the battery black powder and fixed themselves to start biological leaching; however, applying ultrasonic intensification to the system too early might have led to poor leaching. Therefore, we performed ultrasonic intensification once a day from the second day of the reaction, controlling different ultrasonic frequencies, keeping the water bath heated to maintain the incubation temperature in the shaker during the ultrasonic process, and continuing placing it into the shaker after ultrasonic completion. After the ultrasound was completed, the culture was continued in a constant-temperature stacked shaker. The leaching rate of Li, pH, and ORP changes every 24 h was measured to determine the effect of different ultrasonic frequencies on enhancing leaching and, therefore, the length of the leaching cycle. (The pH was measured using a Leici E-201-C composite electrode with a PHS-3C pH meter. For ORP measurement, a Leici 501 composite electrode was used in conjunction with a PHS-3C pH meter.)

##### Ultrasound time

Varying ultrasound durations were explored to optimize intensification without causing detachment of *A. ferrooxidans* from the battery’s black powder surface. The impact of different durations on the enhanced leaching and its effect on the leaching cycle length were evaluated.

#### Exploration of the leaching mechanism

##### Leaching mechanism

A filter bag experiment was conducted to determine the leaching mechanism of A*. ferrooxidans* from LiFePO_4_ batteries. A 0.1 µm pore size filter bag was employed to selectively separate *A. ferrooxidans* (0.5 µm wide and 1 µm long) from the battery black powder. The assessment involved measuring the leaching rate of Li, pH, and ORP changes every 24 h to distinguish between the contact and non-contact leaching mechanisms^38^. If the mechanism is contact-based, it implies that direct interactions between the bacteria and solid particles are necessary for leaching. This would affect how we design bioreactors and pretreatment processes, as ensuring adequate contact between bacteria and solid particles would be essential. On the other hand, a non-contact mechanism suggests that leaching can occur through indirect actions, such as the bacteria releasing soluble factors that facilitate metal dissolution. This might allow for more flexibility in process design.

### Analytical methods

#### Leachate analysis

The leachate analysis consisted mainly of measuring the daily pH and ORP changes during the leaching process using pH and ORP meters at room temperature and measuring the protein concentration changes per hour for the first 5 h of leaching. After the end of the 7-day leaching process, the leaching solution was filtered using a 0.22 µm pore size filter and diluted to a certain multiple, which was measured by ICP-OES, and the leaching rate of Li and Fe was calculated using Formula (1). The graphite in the battery black powder was insoluble and remained in the filter residue; therefore, the influence of graphite in the black powder was not considered in the subsequent experiments^38^.

1 $${LE_{M} = \frac{{C_{L} \times V_{L} }}{{C_{L} \times V_{L} + m_{Z} \times W}} \times 100\% }$$

2 $${W = \frac{{C_{Z} \times 100}}{m}}$$

where LE_M_ is the leaching rate of metal M (%); C_L_ is the concentration of metal in the immersion solution, mg/L; V_L_ is the volume of the leaching solution, L; M_Z_ is the mass of the filter slag (80 °C drying for 24 h after weighing), mg; W is the mass fraction of metal in the filter slag (%); C_Z_ is the concentration of metal in digestion solution, mg/L; and M is the scale number of filter residue, mg.

#### Analysis of filter residue

After the biological leaching process, the remaining filter residue, filtered by the leaching solution, underwent meticulous washing and subsequent drying with deionized water. Analysis of the material composition within the filter residue was performed using XRD. Moreover, alterations in the morphology of the battery black powder surface before and after leaching were observed through scanning electron microscopy (SEM).

#### Determination of protein and *A. ferrooxidans* content

##### Protein content

**Fig. 2:**
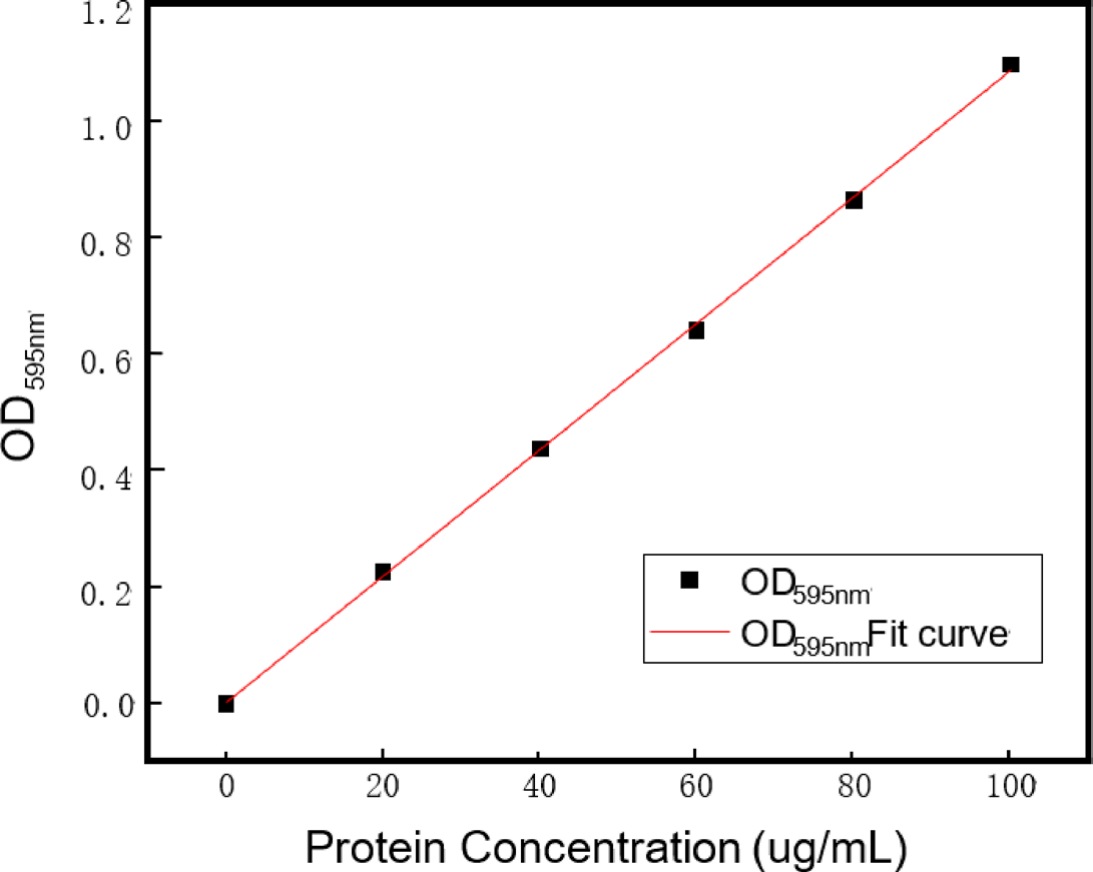
Bradford, method protein standard curve.

Coomassie blue staining was used because of its simplicity, rapid determination, independence from other chemical influences, and accuracy. Before assessing the protein content of the solution, the establishment of a protein standard curve (Fig. 2) was imperative, exhibiting a correlation coefficient R^2^ of 0.9995. This curve served as the basis for the subsequent determination by correlating the absorbance values at A595 (Table 2).

**Table 2:** Protein concentrations versus OD_595nm_.

Protein concentration (µg/mL)	0	20	40	60	80	100
OD_595nm_	0	0.226	0.437	0.641	0.864	1.097

##### *A. ferrooxidans* content determination

Quantification of *A. ferrooxidans* content relied on measuring the protein concentration within the solution, employing the Coomassie blue staining method.

The formula for calculating the *A. ferrooxidans* content is as follows:

3 $$Q = \frac{{Q_{1} }}{2.4}$$

where Q is the content of the *A. ferrooxidans* (mg/g), and Q_1_ is protein content (mg).

## Results and discussion

### Optimal conditions for unapplied ultrasonic enhancement

Under optimal conditions without ultrasound application, the experimental results revealed a lithium leaching rate of 93.1%. These conditions included a solid–liquid ratio of 30 g/L, incubation temperature of 30 °C, oscillation rate of 120 rpm, inoculum volume of 20%, and initial pH of 2.0.

### Effect of ultrasound frequency on leaching by *A. ferrooxidans*

**Fig. 3:**
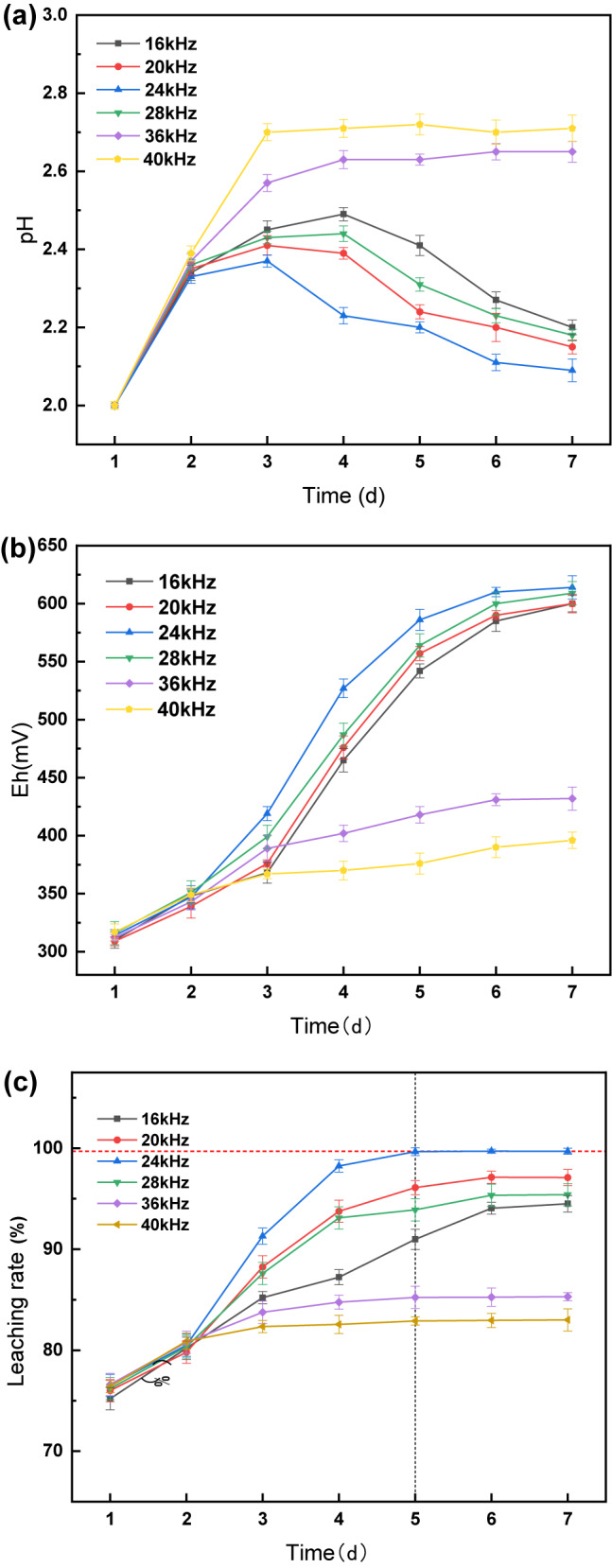
Under different ultrasound frequency conditions changes in pH (**a**), ORP (**b**) and Li leaching rate (**c**) over time.

Leaching experiments were conducted at different ultrasound frequencies, and the results are shown in Fig. 3. Observations from day 1 indicated minimal variation among the groups owing to the absence of ultrasound intensification. Starting on day 2, when ultrasonic intensification was initiated, notable differences emerged. The pH initially increased, which was attributed to the alkalinity of the battery black powder, followed by a decline as H^+^ was produced by *A. ferrooxidans* growth and metabolism. Simultaneously, the ORP increased because of the continuous Fe^3+^ release from the leaching solution.

In the 36 and 40 kHz groups, characterized by a high ultrasound frequency, the pH remained stable without a decreasing trend after increasing to 2.65 and 2.71. The leaching rate stabilized from day 3, with final Li leaching rates of 85.3% and 83%, which were lower than the pre-ultrasound intensification rate. This indicated that ultrasound frequencies above 36 kHz severely impacted *A. ferrooxidans* activity, leading to rupture and death and hindering the bioleaching process.

Conversely, in the 16 and 20 kHz groups with lower ultrasound frequencies, the pH decreased after day 4, reaching the lowest values of 2.20 and 2.15. The ORP values were consistently lower than those of the 24 kHz group, and the Li leaching rate leveled off on days 6–7 at 94.5% and 97.1%, respectively. The 28 kHz group showed a trend similar to that of the 24 kHz group but with less effective ultrasonic enhancement, resulting in a maximum leaching rate of 95.4%. This suggests that ultrasonic frequencies that were too low did not completely remove precipitation, prevented the dissolution layer, and were insufficient to activate the biochemical reactions of *A. ferrooxidans* and chemical reactions promoted by cavitation.

In the 24 kHz group, the pH decreased from day 3, reaching a minimum of 2.09, with the highest ORP value of 614 mV. The Li leaching rate leveled off after day 5, with a notable rate of 99.7%. This demonstrated that 24 kHz ultrasonic intensification provided the optimal conditions for *A. ferrooxidans* activity, enhancing the leaching efficiency and reducing the bioleaching process from 7 to 5 days.

### Effect of ultrasound time on leaching by *A. ferrooxidans*

**Fig. 4:**
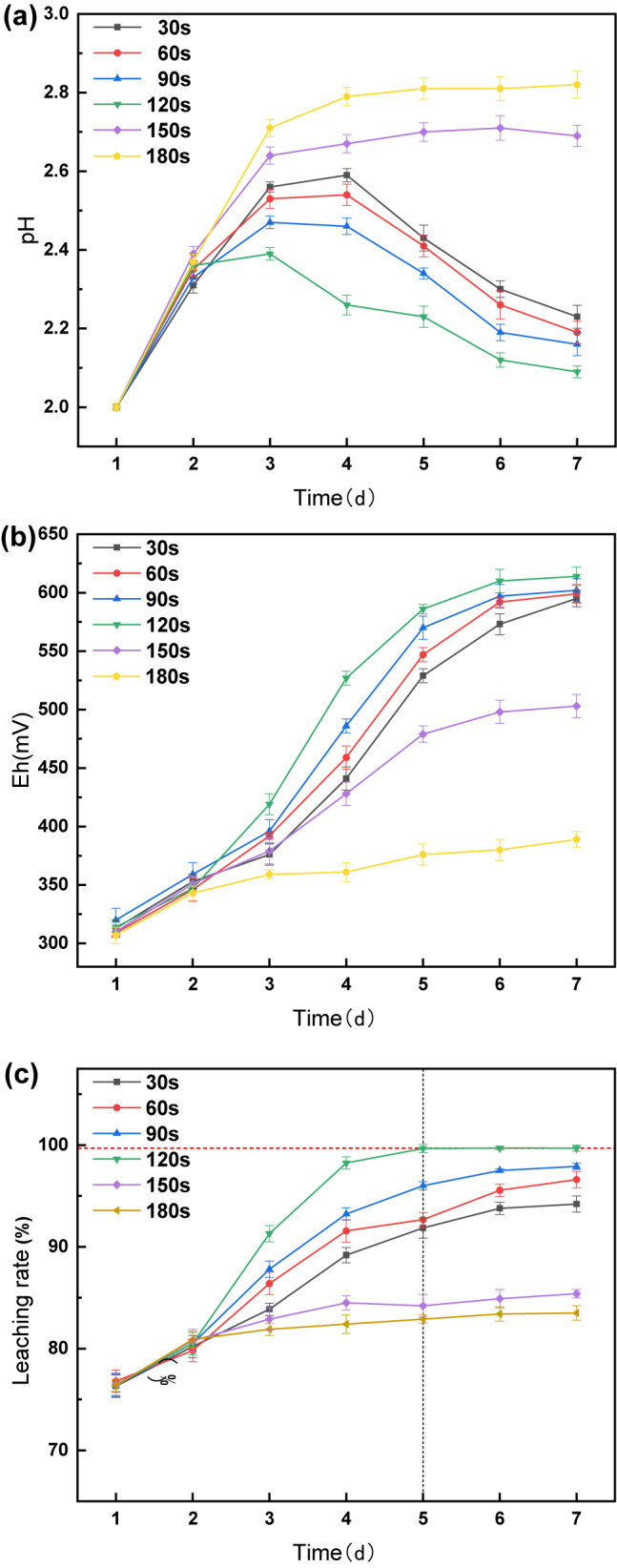
Under different ultrasound time conditions Changes in pH (**a**), ORP (**b**), and Li leaching rate (**c**) over time.

The results of the leaching experiments with varying ultrasound times are shown in Fig. 4. On day 1, without ultrasound enhancement, the pH, ORP, and Li leaching rates showed minimal variation among the groups under consistent incubation conditions.

With the introduction of ultrasound on day 2, noticeable differences emerged between the groups. Similarly, in experiments with different ultrasound frequencies, the pH initially increased due to the alkalinity of the battery black powder, followed by a decline due to the H^+^ produced during *A. ferrooxidans* growth. The continuous release of Fe^3+^ led to an increase in the ORP of the solution, which is consistent with previous observations.

Comparatively, the ultrasound duration was too short in the 30 and 60 s groups. pH decreased later than the 90 and 120 s groups, reaching 2.21 and 2.19, respectively. The ORP was consistently lower than that in the 90 and 120 s groups, peaking at 595 and 599 mV, respectively. Li leaching rates leveled off on days 6–7 at 94.2% and 96.6%, respectively, suggesting that inadequate ultrasound time failed to sufficiently remove surface hindrances and activate biochemical reactions of *A. ferrooxidans*, limiting leaching efficiency.

Conversely, in the 150 and 180 s groups with longer ultrasound times, the pH slowly leveled off at approximately 2.65 and 2.81, with an ORP of only 503 and 389 mV, respectively. Although the Li leaching rate exhibited a slowly increasing trend, reaching 84.2% and 83.1% by day 7, prolonged ultrasound treatment led to *A. ferrooxidans* detachment from the surface of the battery black powder, interrupting the bioleaching process. This resulted in the need for readsorption of *A. ferrooxidans* after ultrasound cessation, which affected the leaching rate.

The 90 s group showed a trend similar to the 120 s group; however, the ultrasound effect was less prominent than the 120 s group. The optimal conditions for the ultrasonic enhancement of *A. ferrooxidans* to leach LiFePO_4_ black powder were identified as 24 kHz and 120 s, reflecting the conditions observed in the experiments with different ultrasound frequencies and achieving the highest leaching rate in the shortest time.

The leaching rates of Li for the enhanced *A. ferrooxidans* cell black powder at different ultrasound frequencies and times are summarized in Table 3.

**Table 3:** Leaching rates of Li under different ultrasound conditions.

Order number	Ultrasound frequency (kHz)	Ultrasound time (s)	Li leaching rate (%)
1	16	120	94.5
2	20	120	97.1
3	24	120	99.7
4	28	120	95.4
5	36	120	85.3
6	40	120	83.0
7	24	30	94.2
8	24	60	96.6
9	24	90	97.9
10	24	150	84.2
11	24	180	83.1

### Filter residue analysis

**Fig. 5:**
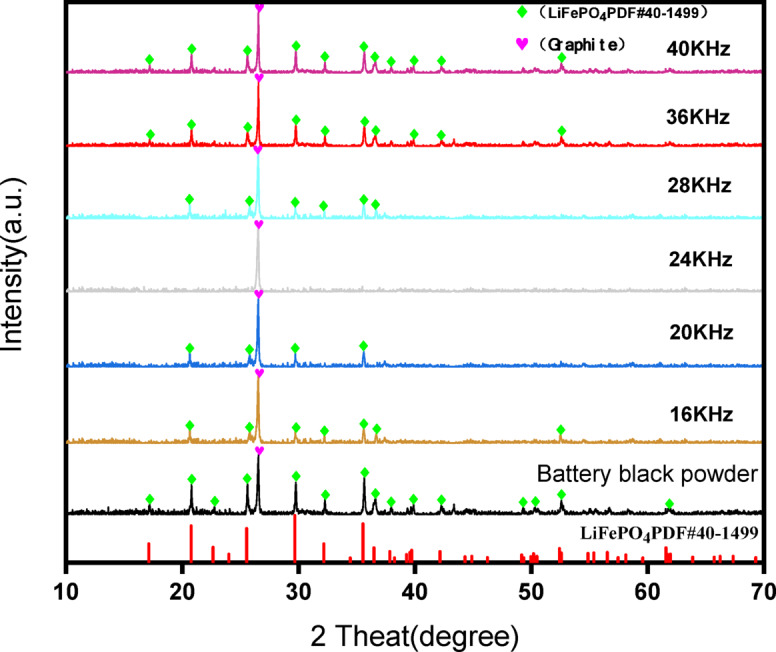
XRD of a filter before and after enhanced leaching under different ultrasonic frequency conditions.

**Fig. 6:**
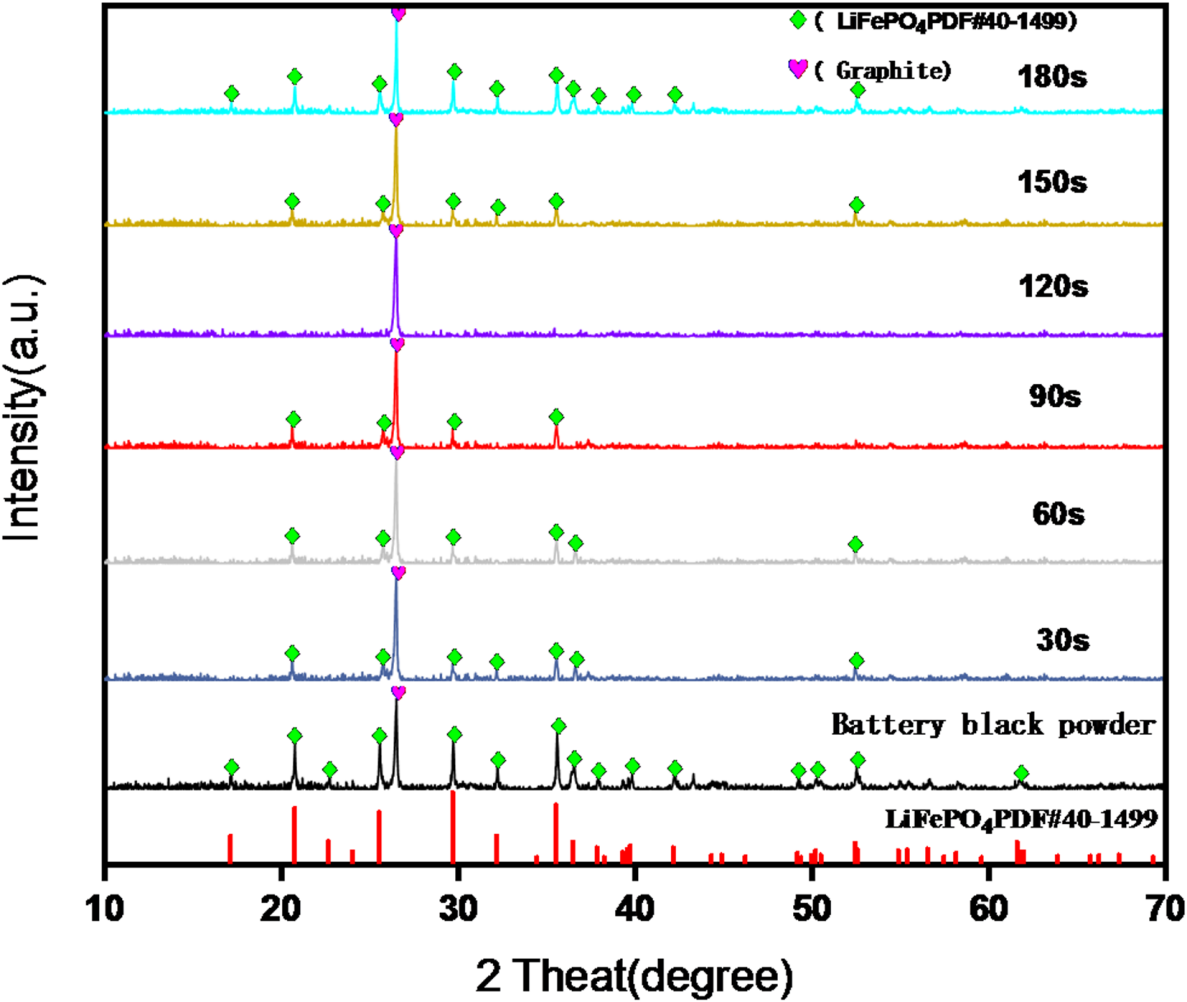
XRD diagram of filter residue before and after enhanced leaching under different ultrasound time conditions.

The XRD patterns of the filter residue at different ultrasound frequencies are shown in Fig. 5, while the XRD patterns of the filter residue at different ultrasound times are shown in Fig. 6.

Figure 5 shows that the diffraction patterns in the filter slag corresponded to the LiFePO_4_ and graphite phases without the emergence of new impurities. At 36 and 40 kHz ultrasonic frequencies, the LiFePO_4_ diffraction peaks were slightly reduced but largely intact. However, at frequencies of 16, 20, and 28 kHz, the LiFePO_4_ peaks notably decreased, and some faint peaks disappeared. Notably, at 24 kHz, the LiFePO_4_ phase was completely eliminated, leaving only a graphite diffraction peak in the filter slag.

Figure 6 shows that the diffraction patterns of the filter residue matched those of LiFePO_4_ and graphite without any additional impurities. At a sonication time of 180 s, the LiFePO_4_ diffraction peak was slightly reduced but was predominantly intact. However, at sonication times of 30, 60, 90, and 150 s, the intensities of the LiFePO_4_ peaks decreased, with some faint peaks disappearing. After sonication for 120 s, the LiFePO_4_ phase was eliminated, leaving only the graphite diffraction peak in the filter residue. These findings align directly with the lithium leaching rate, particularly at 24 kHz and 120 s, where almost all the lithium was successfully leached.

**Fig. 7:**
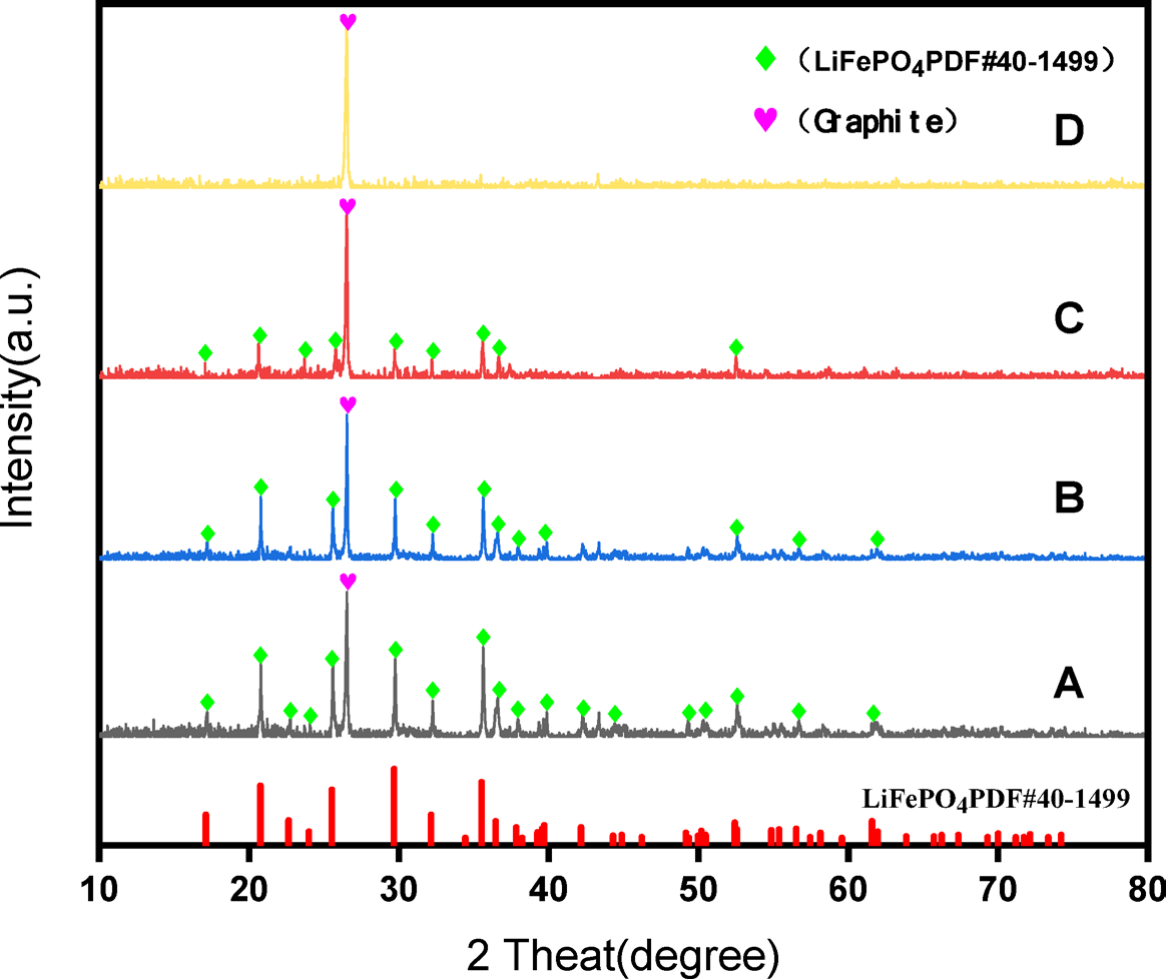
XRD diagram before and after leaching (A: Battery black powder; B: modified 9K medium in blank control experiment; C: Filter residue after bioleaching of battery black powder under optimal conditions; D: Filter residue after ultrasonic enhanced bioleaching of battery black powder under optimal conditions).

Figure 7 shows the sequential progression from group B (acidic conditions) to group C (with *A. ferrooxidans* for bioleaching) and finally to group D (with ultrasonic enhancement). This progression indicated a gradual decrease and eventual disappearance of the LiFePO_4_ diffraction peak, whereas the graphite phase remained consistent throughout. These findings emphasize the essential role of *A. ferrooxidans* and ultrasonic enhancement in facilitating efficient leaching.

**Fig. 8:**
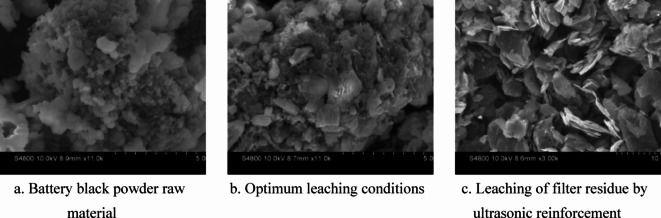
SEM diagram of filter residue before and after leaching [(**a**) Battery black powder raw material; (**b**) Optimum leaching conditions; (**c**) Leaching of filter residue by ultrasonic reinforcement].

Figure 8 shows the SEM images of the filter residue before and after leaching. Image (a) shows the raw LiFePO_4_ battery black powder with a smooth spherical structure. Image (b) reveals that under optimized leaching conditions, severe corrosion occurred on the LiFePO_4_ surface owing to *A. ferrooxidans* action, possibly leading to the incomplete leaching of LiFePO_4_ owing to a flaky graphite structure. Finally, image (c) shows the ultrasonically enhanced leaching residue, where only the flaky graphite structure remained, indicating that LiFePO_4_ is absent.

### Bioleaching mechanism of black powder in LiFePO_4_ batteries

For this experiment, three control groups were established:

*Group A* The battery black powder was wrapped in a filter bag and added to the culture solution for incubation.

*Group B* The battery black powder was enclosed in a filter bag within the culture solution for 72 h, after which the filter bag was removed, allowing direct contact with the culture solution.

*Group C* The battery black powder was added directly to the culture solution for incubation.

**Fig. 9:**
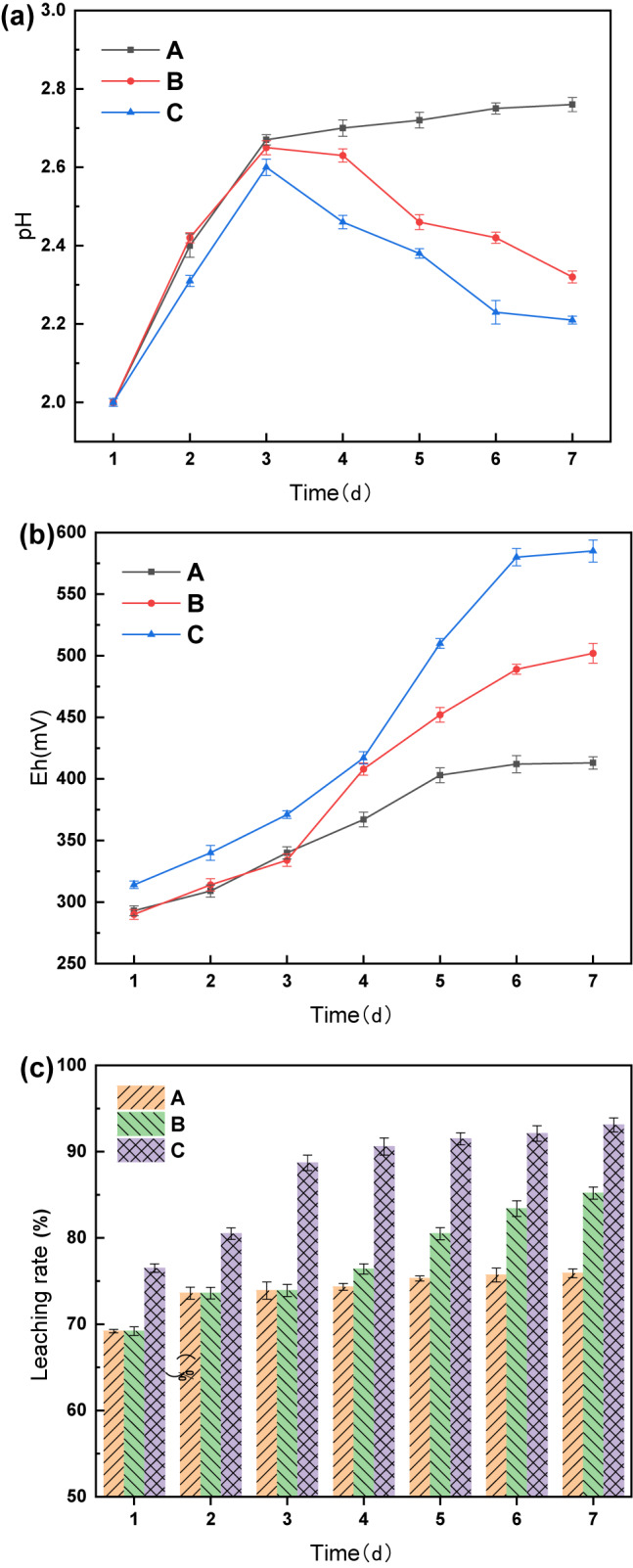
A, B, C group pH (**a**), ORP (**b**) and Li leaching rate (**c**) changes over time.

The changes in the pH and ORP over time are shown in Fig. 9a and b, respectively, and the Li leaching rate is shown in Fig. 9c. Each group contained two parallel samples, and the results were averaged.

In Group A, pH stopped decreasing after an initial increase, reaching a maximum of 2.76. ORP showed the slowest increase, peaking at only 413 mV, with a Li leaching rate of only 75.9%. This indicated a nearly stalled bioleaching process, suggesting that *A. ferrooxidans* could not access the surface of the black battery powder. The waste medium method has a certain leaching effect because the medium contains metabolic products of *A. ferrooxidans*, such as organic and inorganic acids, which can react with metal ions in solid waste to partially dissolve them. However, after filtering out the bacteria, there are no living microorganisms to continue producing these substances, so the concentration of metabolic products gradually decreases, weakening the leaching ability.

Initially subjected to conditions similar to those of Group A for 72 h, Group B showed comparable results. However, after removing the filter bag after 72 h, allowing direct contact between *A. ferrooxidans* and the battery black powder, there was a subsequent decrease in pH, a rapid increase in ORP, and a higher lithium leaching rate of 85.2% over 7 days. This transition highlights a shift in the bioleaching process from limited to direct access, resulting in improved leaching.

Group C exhibited the lowest pH, highest ORP, and highest Li leaching rate because *A. ferrooxidans* was in continuous contact with the battery black powder for 7 days.

Petersen^39^ explained that biological oxidation can occur in solution, within biofilms on mineral surfaces, or a combination of both. Biofilms are a combination of microorganisms (usually multiple species in a symbiotic relationship). The core of bioleaching is the oxidation of Fe^2+^ to Fe^3+^ (Reaction (4)).

4 $$4{\text{Fe}}^{2 + } + {\text{O}}_{2} + 4{\text{H}}^{ + } - \left( {{\text{bio}}} \right) \to 4{\text{Fe}}^{3 + } + 2{\text{H}}_{2} {\text{O}}$$

**Fig. 10:**
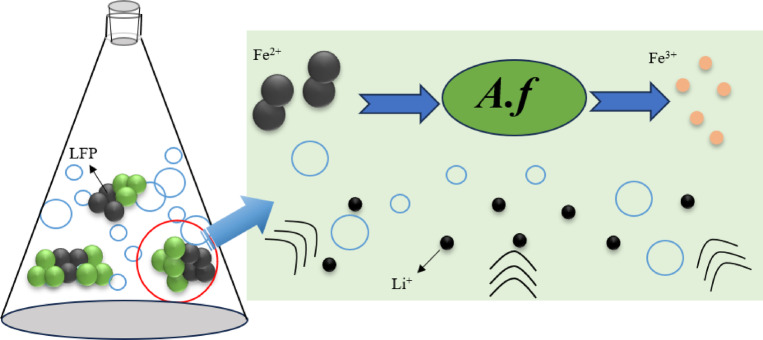
Mechanism of leaching black powder of LiFePO_4_ battery by *A. ferrooxidans.*

The filter bag experiment demonstrated that its presence hindered the bioleaching process, emphasizing the necessity of direct contact between *A. ferrooxidans* and the LiFePO_4_ battery black powder for an effective bioleaching reaction. Consequently, the leaching mechanism of *A. ferrooxidans* on LiFePO_4_ battery black powder was determined to be a contact leaching mechanism. Figure 10 shows a schematic of this mechanism.

### Analysis of adsorption equilibrium and dynamics

The bacterial population within the system remains essentially constant in equilibrium during the first 24 h of bioleaching^40^; therefore, to calculate the content of *A. ferrooxidans* adsorbed on the surface of the battery black powder within the leaching system, only the total *A. ferrooxidans* content of the system minus the free *A. ferrooxidans* content must be calculated.

#### Adsorption behavior of *A. ferrooxidans* on the black powder surface of LiFePO_4_ batteries

*Acidithiobacillus ferrooxidans* cultured to log growth was introduced into the medium at different inoculum levels (ranging from 10 to 30%) along with 30 g/L of battery black powder. The mixture was then placed in a constant-temperature stacked shaker for 15 min to ensure complete mixing. The subsequent cells’ incubation conditions included 30 °C and 120 rpm.

The measurement of *A. ferrooxidans* content commenced at time 0 (total *A. ferrooxidans* content), continuously monitoring the free *A. ferrooxidans* content in the system every 30 min. This measurement was continued for 300 min to calculate the adsorbed *A. ferrooxidans* content.

**Fig. 11:**
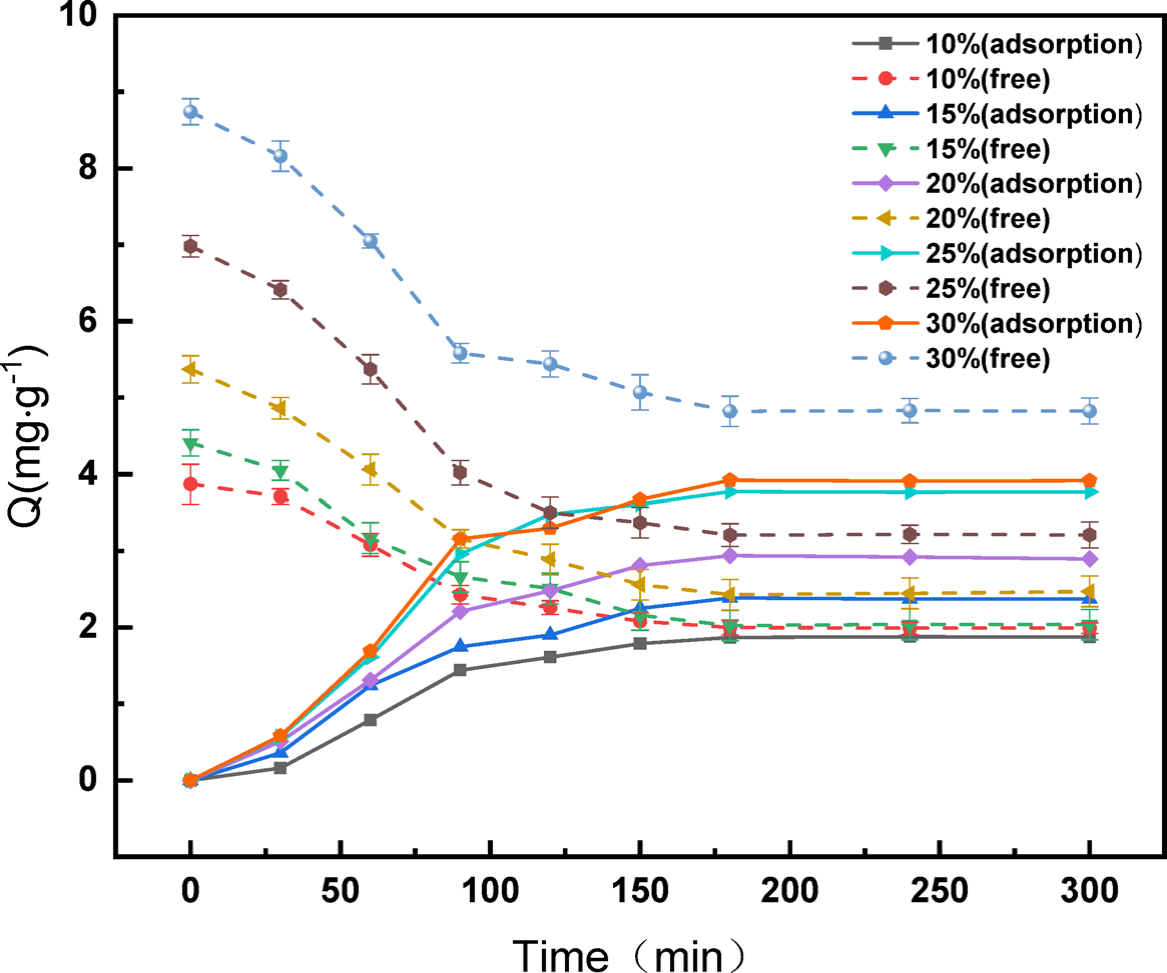
Different inoculations *A. ferrooxidans* Schematic diagram of adsorption balance of LiFePO_4_ battery.

The adsorption of *A. ferrooxidans* followed a similar trend at all inoculum levels. The results are shown in Fig. 11. Initially, adsorption accelerated gradually, slowed after 90–120 min, and stabilized at approximately 300 min, marking the attainment of dynamic equilibrium between adsorbed and free *A. ferrooxidans*. The maximum adsorption capacities of *A. ferrooxidans* were 1.8772, 2.3714, 2.8979, 3.7695, and 3.9155 at 10%, 15%, 20%, 25%, and 30% inoculum levels, respectively. The percentage of adsorbed *A. ferrooxidans* relative to the total *A. ferrooxidans* content ranged from 44.81 to 54.01%. Lower inoculum levels resulted in lower adsorption percentages, owing to the reduced concentration of *A. ferrooxidans* in the solution. This phenomenon correlated with the comparatively lower leaching of lithium observed at the 10% and 15% inoculum levels, as opposed to the higher levels.

At 30% inoculum, the percentage adsorption of *A. ferrooxidans* began to decline, which was attributed to the high *A. ferrooxidans* concentration and insufficient sites available in the solution for cell-black powder adsorption.

#### Model analysis of the adsorption dynamics

The first step in the reaction of *A. ferrooxidans* bioleaching the black powder from LiFePO_4_ batteries begins with the adsorption of *A. ferrooxidans* on the surface of the battery black powder. The adsorption kinetics of *A. ferrooxidans* on the surface of the LiFePO_4_ battery black powder were modeled using the most applied adsorption first-order kinetics and adsorption second-order kinetics models in the field of adsorption by nonlinear fitting of the adsorption content of *A. ferrooxidans* at different inoculum levels^41,42^. The adsorption order and second-order kinetic model equations are as follows.

First-order dynamics of adsorption:

5 $$\frac{dQ}{{dt}} = k_{1} \times \left( {Q_{e} - Q_{t} } \right)$$

Transform conversion:

6 $$Q_{t} = Q_{e} \times \left[ {1 - {\text{exp}}\left( { - k_{1} \times t} \right)} \right]$$

First-order dynamics of adsorption:

7 $$\frac{dQ}{{dt}} = k_{2} \times \left( {Q_{e} - Q_{t} } \right)^{2}$$

Transform conversion:

8 $$Q_{t} = \frac{{k_{2} \times t \times Q_{e}^{2} }}{{1 + k_{2} \times t \times Q_{e} }}$$

where Q_*e*_ is the equilibrium adsorption capacity (mg/g); Q_*t*_ is the adsorption capacity at time t (mg/g); k_1_, k_2_ are the kinetic constants of adsorption First order kinetics and second order kinetics in min^−1^, g min^−1^ min; t is time (min).

**Fig. 12:**
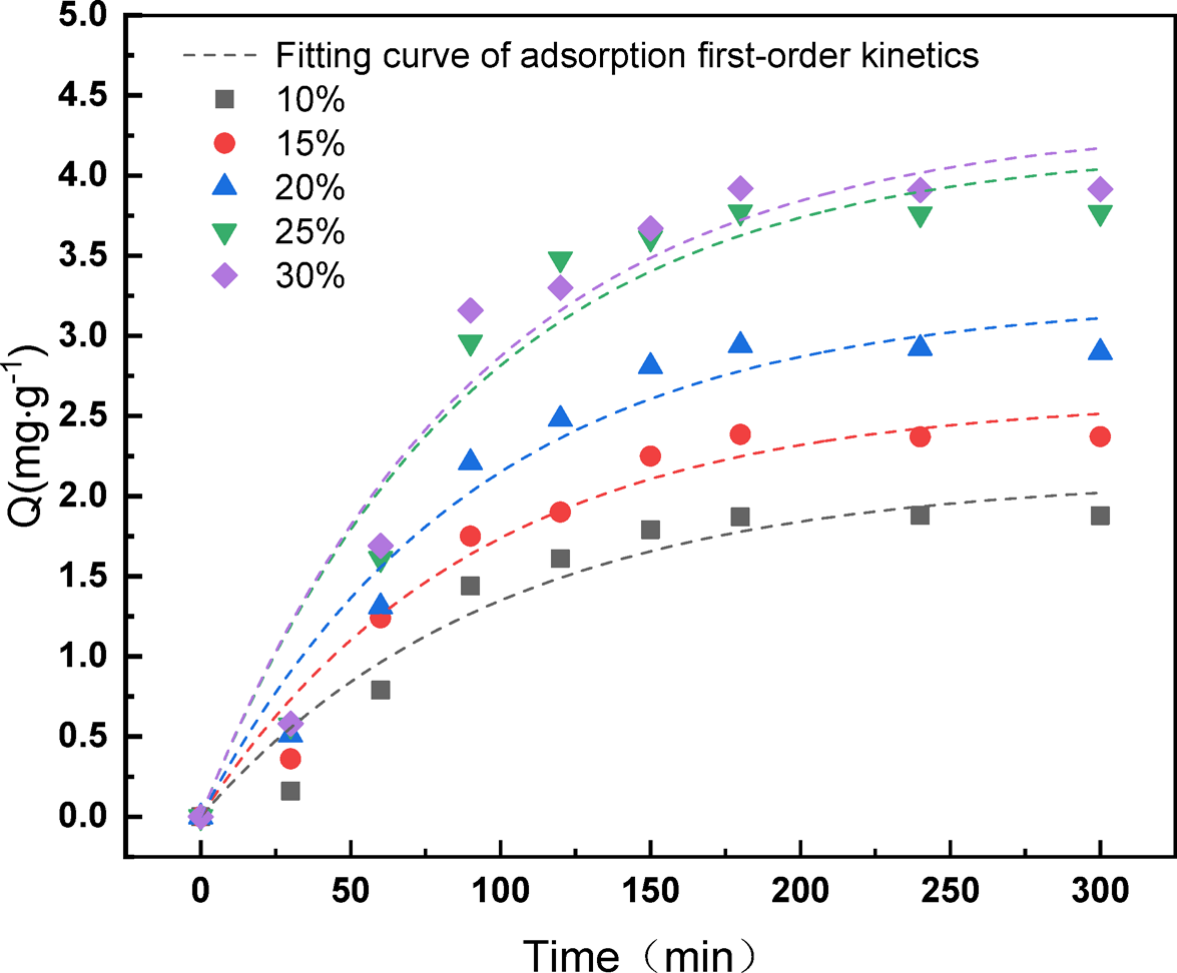
Nonlinear fitting curve of adsorption first-order dynamics of different inoculum levels *A. ferrooxidans.*

**Fig. 13:**
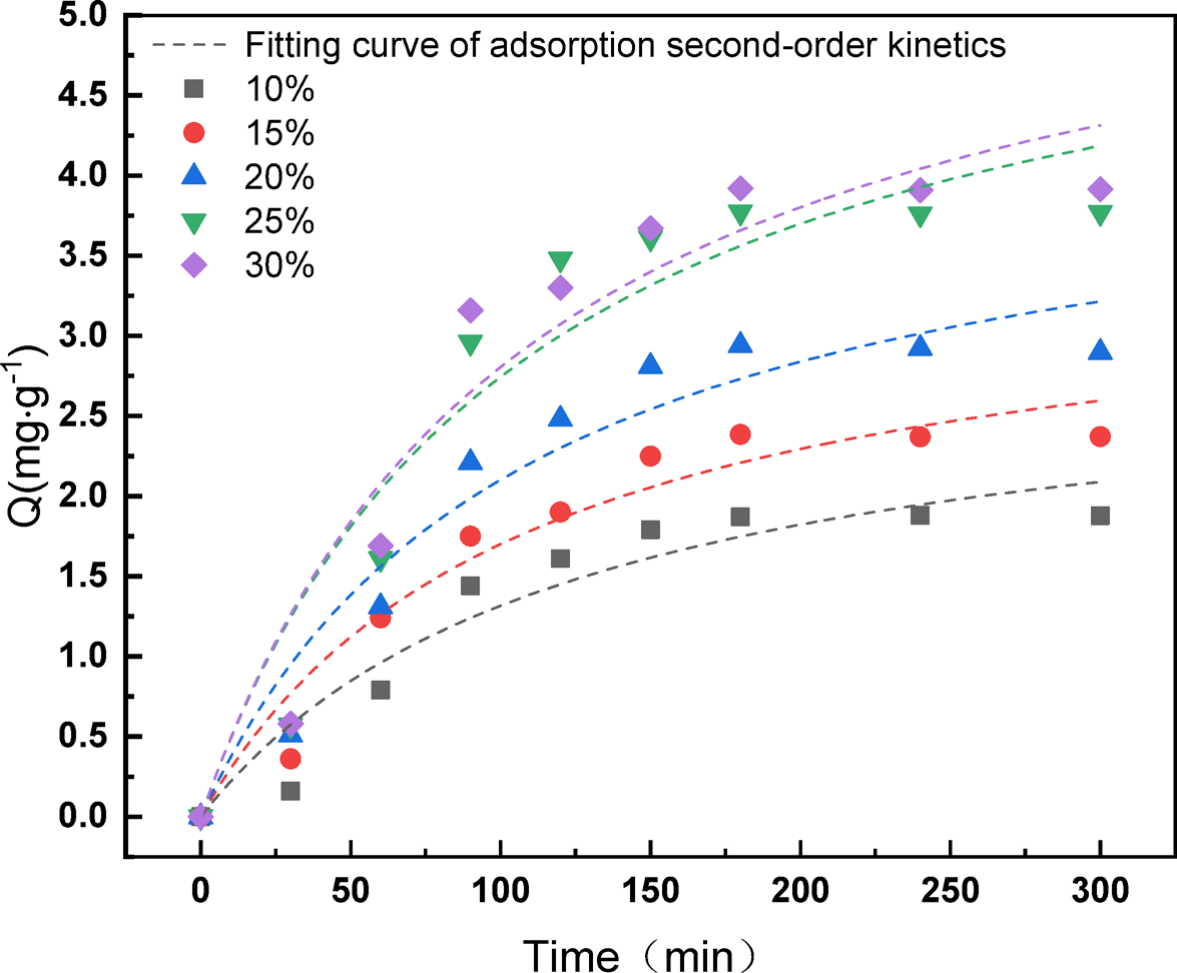
The nonlinear fitting curve of adsorption second-order dynamics of different inoculum levels *A. ferrooxidans.*

Using Origin, first- and second-order kinetics nonlinear fittings were performed for the adsorption content of *A. ferrooxidans*. The results are shown in Figs. 12 and 13, and the fitted first- and second-order kinetic parameters for adsorption are listed in Tables 4 and 5.

**Table 4:** First-order kinetic parameters for the adsorption of *A. ferrooxidans* with different inoculum levels.

First-order kinetics of the adsorption process	10% Vaccination	15% Vaccination	20% Vaccination	Twenty-five percent of the inoculum	30% Vaccination
Q_e_	2.1243	2.6105	3.2308	4.1874	4.3384
k_1_	0.01008	0.01096	0.01096	0.01116	0.01085
R^2^	0.9303	0.9628	0.9571	0.9375	0.9442

**Table 5:** Second-order kinetic parameters for the adsorption of *A. ferrooxidans* with different inoculum levels.

First-order kinetics of the adsorption process	10% Vaccination	15% Vaccination	20% Vaccination	Twenty-five percent of the inoculum	30% Vaccination
Q_e_	2.9561	3.5229	4.3712	5.6752	5.9009
k_2_	0.00272	0.00265	0.00212	0.00165	0.00154
R^2^	0.9102	0.9452	0.9367	0.9147	0.9243

The correlation coefficients R^2^ from the nonlinear fitting of the adsorption first-order kinetics were greater than R^2^ from the adsorption second-order kinetics, the first-order kinetics constants k_1_ were greater than the second-order kinetic constant k_2_, and the Qe values obtained from the fitting of the adsorption first-order kinetics were closer to the adsorption equilibrium capacity derived from actual measurements (Tables 4 and 5). It can be concluded that the adsorption first-order kinetics were more consistent in describing the adsorption behavior of *A. ferrooxidans* on the black powder surface of LiFePO_4_ batteries than the adsorption second-order kinetics.

## Conclusions

This study aimed to assess the practical feasibility of bioleaching LiFePO_4_ batteries using *A. ferrooxidans*, specifically without the addition of FeSO_4_. Parameters investigated included a solid/liquid ratio of 30 g/L, an incubation temperature set at 30 °C, a shaking rate of 120 rpm, 20% inoculum concentration, and an initial pH of 2.0.

A system was established to investigate the ultrasonically enhanced leaching of black powder from LiFePO_4_ batteries using *A. ferrooxidans*. This system demonstrated increased leaching rates and reduced leaching times. Optimization of the bioleaching conditions showed that under the optimum parameters, including ultrasonic enhancement (24 kHz ultrasound once every 24 h for 120 s over a 5-day leaching period), the bioleaching rate of lithium increased to 99.7%.

These results demonstrated the ability of *A. ferrooxidans* to produce sulfuric acid and oxidize Fe^2+^ in LiFePO_4_ to Fe^3+^, thereby indirectly leaching metals from lithium-ion batteries (LIBs). This study highlights the practical potential of bioleaching to recover valuable materials from LIBs, which holds considerable promise for sustainable resource recovery.

## Data Availability

The datasets used and/or analysed during the current study available from the corresponding author on reasonable request.
